# Using Public Health Approach to make our programme logic 

**Published:** 2019-08

**Authors:** Shumei Wang

**Affiliations:** ^*a*^ School of Public Health, Fudan University, Shanghai, China.

## Abstract

Among the seven indicators in international safe community, it is one of the core elements to solve the injury problem in the community in a project-based manner. The main purpose of the International Safe Community Certification Center promoting model projects and excellent projects to the world, is to provide good example and reference for the community. How to identify the main risk in the community? How to take appropriate intervention measures according to the specific conditions of the community? How to maximize the effectiveness of the project and effectively solve the major injury problems of the community? How to evaluate the programme? Those are the challenges many communities are facing.

Fortunately the public health approach which includes four steps provides the best tool for communities to implement effective injury prevention programs. (1) Surveillance – the community need to know and understand the problems in their community. Through surveillance it is possible to determine the magnitude and characteristics of the problem, and can include factors such as when, where, to whom and how the injury happened. (2) Risk factor identification –the community need to know and understand the causes of the problems. Through risk factor identification it is possible to determine which factors increase the risk of injury or disability and which factors are potentially modifiable. (3) Develop and evaluate interventions –the community need to know and understand what works? By this step the community can develop intervention with set measures to assess if the injury problem was addressed with using information gained from risk factor identification to develop and pilot-test or evaluate interventions. (4) Implementation –the community need to know and understand how it is done. This last step aims to implement the most promising interventions on a broader scale.

The lack of logic in injury prevention programs is one of the most common problems during the safe community certifying process. There are often inconsistencies between the project's background, the interventions adopted, and the assessment indicators. The application of public health approach can effectively help the community. Of course, communities can collaborate with professional institutions or universities, to work with public health professionals, which is one of the effective ways to improve the quality of community injury prevention programs.

**Keywords:**

Public Health, Safe Community, Injury Prevention

